# Automotive braking is a source of highly charged aerosol particles

**DOI:** 10.1073/pnas.2313897121

**Published:** 2024-03-11

**Authors:** Adam E. Thomas, Paulus S. Bauer, Michelia Dam, Véronique Perraud, Lisa M. Wingen, James N. Smith

**Affiliations:** ^a^Department of Chemistry, University of California, Irvine, CA 92697

**Keywords:** brake wear particles, particle charge state, nonexhaust emissions

## Abstract

Although the last several decades have seen a dramatic reduction in emissions from vehicular exhaust, nonexhaust emissions (e.g., brake and tire wear) represent an increasingly significant class of traffic-related particulate pollution. Aerosol particles emitted from the wear of automotive brake pads contribute roughly half of the particle mass attributed to nonexhaust sources, while their relative contribution to urban air pollution overall will almost certainly grow coinciding with vehicle fleet electrification and the transition to alternative fuels. To better understand the implications of this growing prominence, a more thorough understanding of the physicochemical properties of brake wear particles (BWPs) is needed. Here, we investigate the electrical properties of BWPs as emitted from ceramic and semi-metallic brake pads. We show that up to 80% of BWPs emitted are electrically charged and that this fraction is strongly dependent on the specific brake pad material used. A dependence of the number of charges per particle on charge polarity and particle size is also demonstrated. We find that brake wear produces both positive and negative charged particles that can hold in excess of 30 elementary charges and show evidence that more negative charges are produced than positive. Our results will provide insights into the currently limited understanding of BWPs and their charging mechanisms, which potentially have significant implications on their atmospheric lifetimes and thus their relevance to climate and air quality. In addition, our study will inform future efforts to remove BWP emissions before entering the atmosphere by taking advantage of their electric charge.

## Significance

The coming decades promise a transition from internal combustion engines to electric, and with it a greater relative contribution of nonexhaust sources to urban air pollution. A chief concern is particles generated from automotive brake wear, which have adverse impacts on health and the environment. Our study reports on the electrical properties of brake wear particles, demonstrating that up to 80% of these particles are electrically charged. We show evidence for surprisingly high numbers of elementary charges per particle and report on this number’s dependence on particle size and charge polarity. These findings suggest that control strategies that exploit the unique electrical properties of brake wear particles can be highly effective in mitigating this key emerging source of pollution.

Aerosol particles derived from traffic emissions are considered a dominant source of particulate matter (PM) pollution in urban environments (1). Brake wear particles (BWPs) are known to contribute significantly to these emissions and to traffic pollution overall, comprising up to 55% of nonexhaust PM_10_ mass (2), or as much as 21% of total traffic-related PM_10_ mass (3–5). It is almost certain this contribution will grow in the coming decades, considering the further adoption of alternative fuels and the electrification of the global vehicle fleet (6). This emphasizes the need for further research into the role of nonexhaust emissions in general, and brake wear emissions in particular, in order to understand potential impacts and to inform future regulatory efforts. Experimental studies have demonstrated some of the potential health effects of inhaling BWPs across a broad range of sizes (10 nm to >2.5μm), including the induction of pulmonary inflammation in animals and oxidative stress in lung cells (7–11). Due to their abundance, these particles may prove to have important implications for regional climate as well, though this has yet to be rigorously explored.

Despite their emerging importance, a complete picture of the composition and physical characteristics of BWPs is currently lacking. BWPs are generated at the contact area between the brake pad and rotor or drum from the combination of abrasion, producing primary wear particles, and heating, producing low-volatility vapors that nucleate into nanometer-sized particles (12). It is estimated that 35 to 55% of BWPs become airborne (13, 14). Size distributions of BWPs have been extensively investigated, principally as generated using a brake dynamometer (15, 16). Though distribution characteristics seem to vary widely with the specific braking cycle employed and pad material tested, one or several submicron modes are commonly observed alongside a larger coarse mode centered around particle diameters between 1.0 and 2.0 μm. The coarse mode has been attributed to abrasion of pad material while the origin of the smaller modes remains somewhat contentious, although a nanometer-sized mode has been consistently linked to surpassing a certain critical temperature of the pad material used (17–21). Due to the frictional process by which BWPs are generated, it is reasonable to suspect that some of these particles might be electrically charged. The presence of charges on aerosol particles has been shown to enhance coagulative growth (22–24) and modulate transport properties (25, 26). With regard to human health, several studies have shown that surface charges may enhance particle deposition onto lung airways (27–30), although this is still an area of active research (31). Brake pads have been studied as a tribological surface (32), and an electrostatic precipitator was recently proposed as a possible means by which to reduce primary BWP emissions (33). However, to the best of the authors’ knowledge, a study on the electrical charge characteristics of BWPs has yet to be reported and many open questions remain including the number of elementary charges per particle (hereafter referred to as charge state), ratios of charged-to-neutral particles, and the relationships between charge state, braking mechanisms, and particle size/composition.

Here, we present measurements of the electrical charge characteristics of BWPs, as generated experimentally from a disc brake using a custom-built brake dynamometer system. The emissions from two common types of brake pad formulations (semi-metallic and ceramic) were investigated. We examine the fraction of BWPs that are electrically charged and probe their electrical charge state as a function of particle size and braking conditions. We show that moderate braking of either brake pad tested generates charged particles of both polarities and that up to 80% of BWP can be electrically charged. We demonstrate how many of the BWPs measured are multiply charged in the diameter range of 10 to 1,000 nm, with one BWP containing as many as 30 elementary charges.

## Results

### Brake Wear Particle Generation and Size Distributions.

**Fig. 1.**
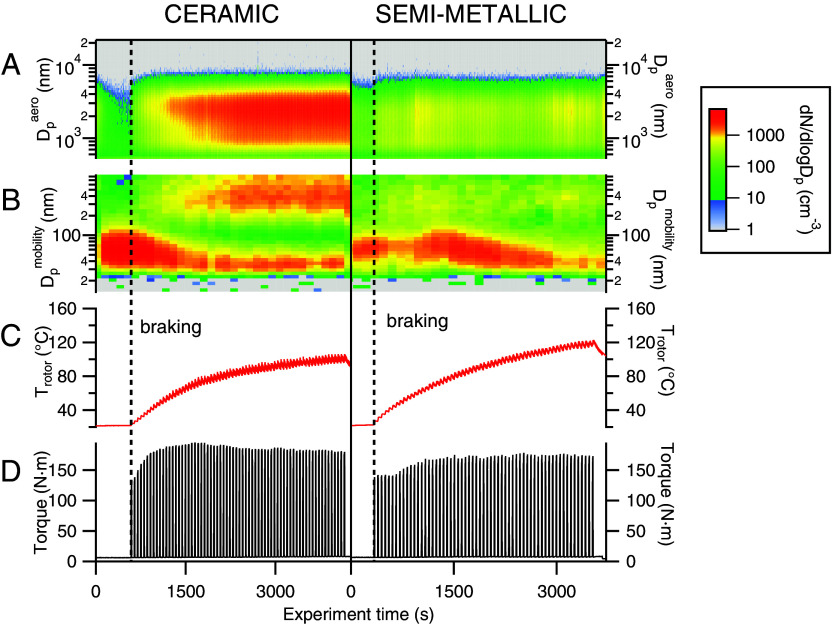
Dynamometer braking conditions and BWP size distributions during experiments with ceramic (*Left*) and semi-metallic (*Right*) brake pads. BWP size distributions were collected from (*A*) an APS, measuring over the aerodynamic diameter range ($D_{p}^{\mathrm{aero}}$) 500 to 22,000 nm and (*B*) from an SMPS, measuring over the electrical mobility diameter range ($D_{p}^{\mathrm{mobility}}$) 10 to 900 nm. Braking state variables (*C*) brake rotor temperature and (*D*) braking torque.

BWP generation was simulated using a custom-built brake dynamometer facility (*Materials and Methods*, Section 1) consisting of a brake rotor rotated at a constant speed (173 rpm) and a hydraulically actuated brake caliper mounted on a rotational torque sensor. Braking was applied using a series of square wave hydraulic pulses over a period of 1 to 2 h. BWP size and number concentrations were monitored across a wide particle diameter range (10 to 22,000 nm) to provide a comprehensive picture of the particle size distribution for each experiment. Fig. 1 shows the BWP size distributions as measured by a scanning mobility particle sizer (SMPS, Fig. 1*B*) and an aerodynamic particle sizer (APS, Fig. 1*A*) as well as the braking state variables (torque and brake rotor temperature) during the first hour of a typical experiment for both ceramic (*Left*) and semi-metallic (*Right*) brake pads, where t = 0 s is the time when the rotor begins spinning at 173 rpm. The brake pressure and temperature of the chamber are provided in *SI Appendix*, Fig. S2. For the same braking cycle (brake fluid hydraulic pressure 1,400 kPa, 8 s brake pulse, 45 s cruising time), use of semi-metallic brake pads resulted in a higher rotor temperature compared to the ceramic pads, while also producing fewer particles overall. Both brake pads generated a multi-modal particle size distribution consisting of at least two modes: an ultrafine (≤100 nm in diameter) mode and a coarse mode centered at an aerodynamic diameter of 2.5 μm. Ultrafine particles appeared even when the rotor was spinning before the onset of braking. This is consistent with previous studies that have reported particle generation due to the residual drag from the brake rotor brushing against the brake pad, resulting in the detachment of wear particles from prior use accumulated along the pores of the pad surface (19, 34–36). Over the course of the experiment, the distribution was dominated by the larger coarse mode of freshly generated particles, most clearly demonstrated in the ceramic brake experiment. Although the number and position of BWP modes observed vary as previously noted, a mode around 350 nm has specifically been reported using different measurement techniques (37, 38), possibly indicating the presence of a third mode in our distributions in addition to the ultrafine and coarse modes. It should be noted however that the presence of multiply charged particles with diameters larger than 900 nm could be contributing to the magnitude of this mode, as such particles would not be charge-corrected with our inversion method. In addition to illustrating the dynamic nature of BWP formation, these results also highlight the differences in particle generation between different brake pad types.

### Charged Particle Fractions and Distributions.

**Fig. 2.**
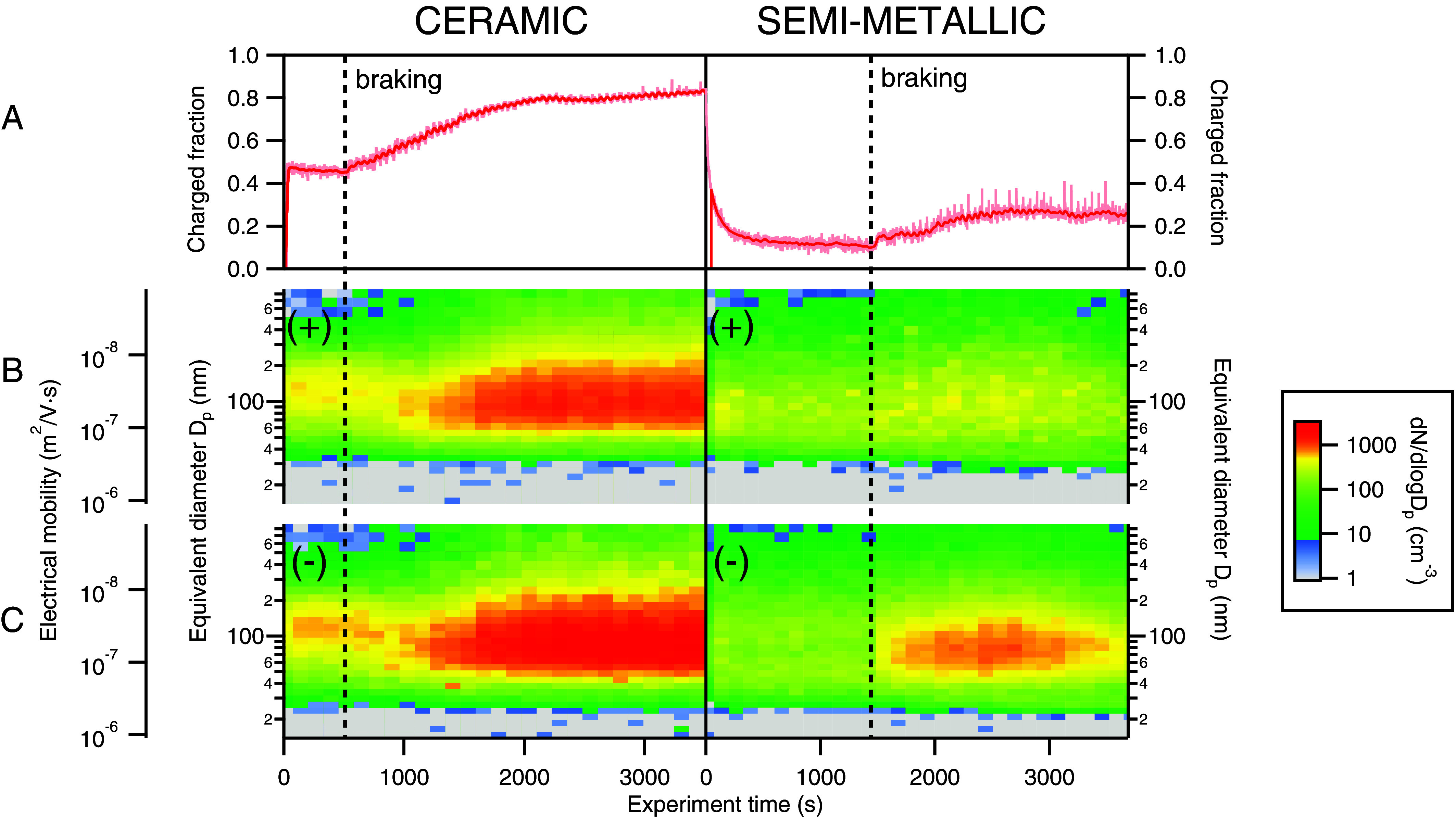
(*A*) Charged particle fractions for experiments with ceramic (*Left*) and semi-metallic (*Right*) brake pads. Charged particle fractions shown in red are smoothed data while the variation in the raw data is shown in a lighter shade. The delimited lines mark the initiation of braking. (*B* and *C*) SMPS electrical mobility distributions of (*B*) positive and (*C*) negative BWPs for both brake types.

The fraction of electrically charged BWP concentration to total BWP concentration was measured with two condensation particle counters (CPCs), one directly sampling the total concentration and the other downstream of an electrostatic precipitator that captured all charged species (*Materials and Methods*, Section 2). Both brake types tested generated charged particles, but with distinct differences in charged fraction. Fig. 2*A* shows the charged fraction over the course of the first hour of experiments for both brake pad types. For the ceramic pads, we observed that spinning the rotor resulted in a 46±1.4% charged fraction (±1 SD), indicating that brake drag generates charged particles during cruising (i.e., no braking) conditions. A similar phenomenon was observed for semi-metallic pads, although the fraction decayed to a much lower value of 11±1.5% during cruising. With the ceramic pads, the fraction started to increase as braking commenced, with spikes coinciding with the onset of each braking event (see *SI Appendix*, Fig. S3 for the full dataset for each experiment). The fraction continued to climb throughout the first half hour of the experiment but then leveled off at a charged fraction of 81±1.5%, a fraction maintained throughout the rest of the experiment. A similar temporal behavior was observed for semi-metallic brakes, albeit with a lower steady-state fraction reached of 25±2.7%.

To examine the distribution of charged BWPs across both polarities, two SMPS systems sampling in parallel were configured so that only ambient charged positive or negative particles could be measured (*Materials and Methods*, Section 3). Fig. 2 *B* and *C* show the electrical mobility distributions of positively and negatively charged BWPs, respectively, from ceramic and semi-metallic brake pads. Both brake types generated positively and negatively charged particles with a wide range range of electrical mobilities. For both pads, braking produced more negatively charged particles. This bias toward the negative polarity is consistent with previous work investigating the emission of charged particles from various materials upon tribological (i.e., frictional) contact, including emissions from ceramic wear surfaces (39, 40). Though the charging mechanism is not completely understood, it is known that electrons can be emitted from frictional contact, a process driven by the excitation of surface electrons on materials with low work functions such as metals (41, 42). It is therefore reasonable to suspect that emitted electrons could ionize surrounding air molecules or directly interact (43) with BWPs to impart electrical charge. In addition, the greater diffusivity of electrons compared to positive ions could also potentially explain the bias toward negative particle charges (44). Compared to the ceramic pads, the semi-metallic brake pads produced fewer charged particles across both polarities, in agreement with our observations of a lower charged fraction (Fig. 2*A*) and fewer total measurable particles (Fig. 1 *A* and *B*) for this pad formulation. These differences in particle generation and charged aerosol properties between the two brake pad types are likely due to morphological differences on the brake pad surface (45) as well differences in composition, specifically in metals (46). Differences in the electronic work functions of the materials used in each brake pad could drive differences in the amount of charge that accumulates on the pad surface. Such a difference is used to describe the triboelectric effect or static electricity (47). Despite these differences, it is intriguing that the distributions of negatively charged particles were centered at a mobility diameter of ∼ 75 nm (or an electrical mobility of $4.5\times 10^{-8}$ m^2^ V^−1^s^−1^) for both brake types during the period of steady-state generation, while the positively charged particles were centered at ∼ 85 nm ($3.6\times 10^{-8}$ m^2^ V^−1^s^−1^), suggesting a common generation mechanism.

### Brake Wear Particle Charge States.

**Fig. 3.**
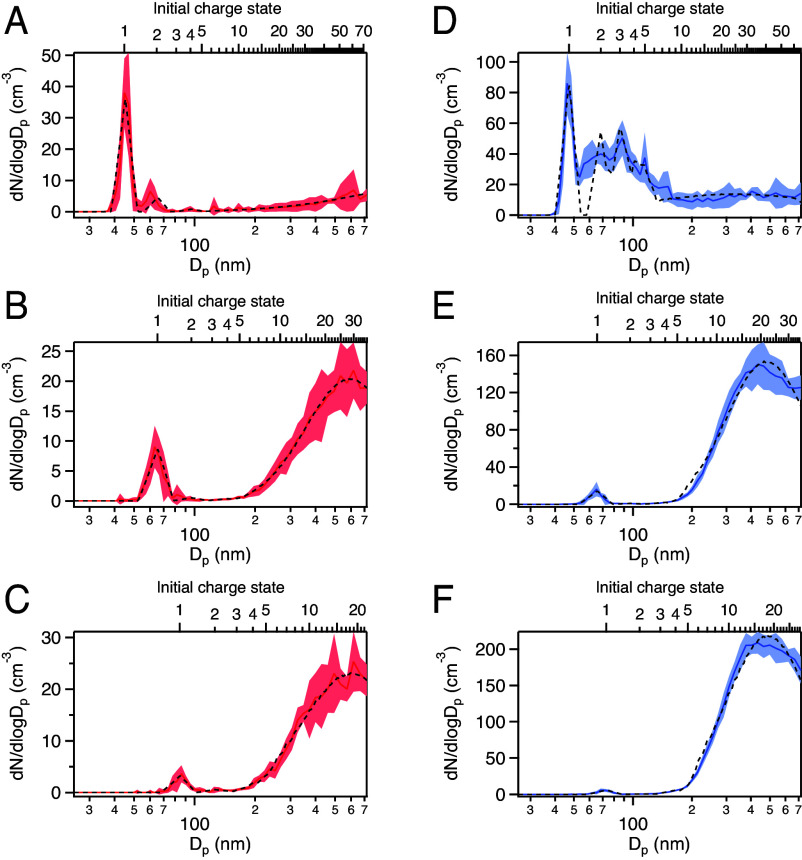
Charge state distributions for BWPs emitted from ceramic brake pads. Distributions of positively charged particles (*Left* column, in red) are shown for particles with selected mobility diameters of (*A*) 45 nm, (*B*) 65 nm, and (*C*) 85 nm. Distributions of negatively charged particles (*Right* column, in blue) are shown for mobility diameters of (*D*) 45 nm, (*E*) 65 nm, and (*F*) 73 nm. The dashed lines indicate the model results. Shaded areas correspond to the SD of multiple measurements.

The electrical charge state of BWPs was investigated using tandem differential mobility analysis (TDMA; *Materials and Methods*, Section 3). In brief, particles with an equivalent singly charged mobility diameter (referred to hereafter as simply “mobility diameter”) ranging from 35 to 95 nm were selected with a first DMA and then neutralized and analyzed for charge state with a second DMA. TDMA has previously been adopted to investigate the charge state of aerosols generated from motor vehicle exhaust (48), as well as ambient aerosols measured near freeways (49). Fig. 3 shows representative neutralized particle size distributions with corresponding charge state measured for ceramic brake pad BWPs. Distributions for all selected mobilities measured for both brake types are provided in *SI Appendix*, Figs. S4 and S5. In general, as we increased the mobility diameter of BWPs measured, we observed that the number of singly charged particles decreases. As exemplified in the positive case, most of the 45 nm charged particles were singly charged (Fig. 3*A*), since the re-neutralized distribution consisted mostly of a peak at 45 nm. In contrast, the re-neutralized distribution for 65 nm charged particles (Fig. 3*B*) mainly featured larger particles and a small peak at 65 nm, indicating that most of the particles measured at this mobility were actually multiply charged. At the mobility diameter corresponding to the peak concentration for positively charged particles (85 nm as shown in Fig. 2*B*), larger particles dominated the size distribution (Fig. 3*C*) indicating that multiply charged particles dominated at this mobility. The TDMA data also provide an explanation for the minimum in the particle size distribution at 100 nm (Fig. 1*B*): The singly charged population decreased at diameters < 100 nm and the multiply charged population were highly charged with mobility diameters > 200 nm. A similar trend was observed for negatively charged particles (Fig. 3*D*–*F*), where the singly charged peak is almost undetectable near the peak concentration for negatively charged BWPs (73 nm mobility diameter, Fig. 2*C*).

Unlike charged particles produced during combustion (48, 50), it is apparent that charged BWPs are not in charge equilibrium, with most particles larger than a mobility diameter of 50 nm having 10 to 30 elementary charges. The average charge state of BWPs measured was calculated by fitting each charge state distribution to a model function (*Materials and Methods*, Section 4). The results are shown in *SI Appendix*, Fig. S7. For the mobility range tested, the average charge state varied between 1 and over 30 elementary charges per particle, depending on the particular mobility measured. Intriguingly, the highest charge states calculated for positively charged particles from either brake pad type exceeded those calculated for negatively charged particles. In the ceramic brake experiments, for example, the highest average charge states observed were 38 ± 1.0 and 26 ± 0.9 for positive and negative 55-nm mobility diameter particles, respectively. Comparing between brake pad types, the charge states observed from the ceramic brake pads were higher than those observed from the semi-metallic pads, specifically for the positively charged particles (e.g., the average charge of a ∼65-nm mobility diameter particle from ceramic brakes was 30 ± 0.4 but 23 ± 3.3 from semi-metallic brakes). This might be an effect of the differences in pad composition but may also be a consequence of the higher torques achieved for the ceramic pads during these experiments. It should be noted that although the brake pneumatic pressure was not changed for each experiment, the resulting torque and rotor temperature were variables that depend on complex factors such as pad history, chamber temperature and RH, and pad and rotor surface morphology (51). Despite the observed differences, it is remarkable how similar the relationship is between average charge state and equivalent mobility diameter across both brake types and polarities, resembling log-normal distributions with maxima located around 50 to 70 nm. Similar to the charge distribution measurement with SMPS, this similarity also suggests a common mechanism is inherent to the braking action.

**Fig. 4.**
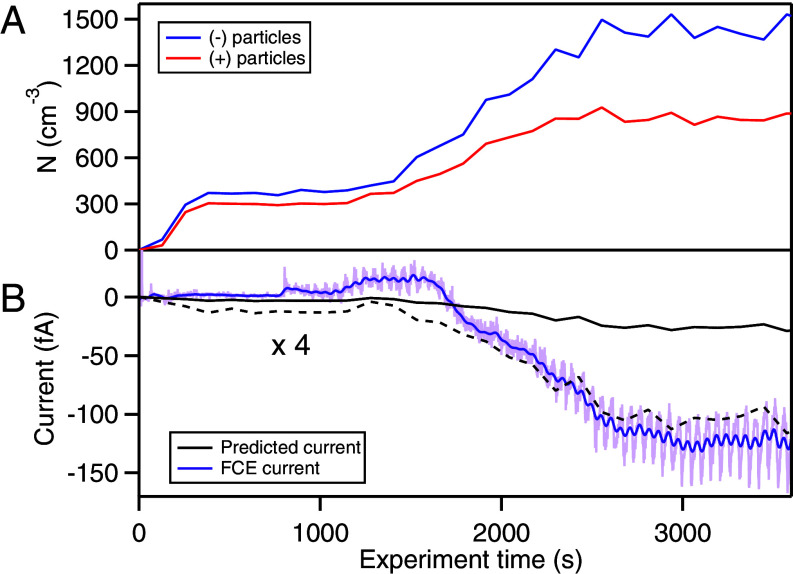
(*A*) Concentrations of negatively (blue) and positively (red) charged particles and (*B*) aerosol currents for ceramic brake pads. Predicted current (solid black) obtained from SMPS and TDMA measurements is plotted with an amplified version (dashed black) to compare the waveform against the FCE current (purple). The light purple trace indicates the raw signal from the FCE while the dark purple line is smoothed data.

Although our observations captured the charge states of BWPs with measurable electrical mobilities, we could not probe the charge states of BWPs larger than the SMPS scanning range with the adopted methodology. To assess the extent to which our observations account for the total number of charged species generated by braking, we compared the aerosol current predicted by our tandem differential mobility analysis to a current measured using a Faraday cup electrometer (FCE), which operated in parallel with the electrical mobility distribution measurements. The predicted current was obtained by applying the model fits from our average charge distributions (*SI Appendix*, Fig. S7) to the electrical mobility data (*Materials and Methods*, Section 4). The results of this comparison for ceramic brakes are shown in Fig. 4, while the results for semi-metallic brakes are shown in *SI Appendix*, Fig. S9. For both brake pad types, our prediction could only account for a fraction of the current observed with the FCE, on average only accounting for 26±11% of the FCE current from ceramic pads, and 18±8.5% for the semi-metallic pads. This implies the existence of charged species with sizes outside the range that can be characterized by TDMA. To investigate the presence of micron-sized charged particles, we also measured the charged particle fraction with the APS (*SI Appendix*, Fig. S10). Although a quantitative fraction cannot be obtained with this measurement, it is apparent from our data that many BWPs with aerodynamic diameters larger than 1,000 nm are also electrically charged. To characterize the charge state of these larger species, an area of future work will focus on coupling a mobility classifier to a micron-particle sizing instrument, such as an APS. As noted previously, it is also reasonable to suspect that gaseous ions and electrons are released during braking events, a well-known consequence of tribological abrasion between metals (52), and are thus also contributing to the FCE current.

### Atmospheric Implications.

The presence of electric charges on aerosol particles in the atmosphere has received recent attention as a property of potentially far-reaching climatic significance. Electrical charges have been shown to enhance the growth of newly formed particles in the atmosphere (53–55), a phenomenon attributed in part to enhanced collision rates with ions of the opposing polarity (56). Coagulation rates with larger particles are also enhanced (23, 24), and the growth rates of cloud condensation nuclei with multiple charges are higher compared to electrically neutral nuclei (57), thereby promoting cloud droplet activation. Particles with many charges (30+) are observed in the atmosphere at the top of thunderclouds, for instance, where it has been demonstrated they can provide a pathway to ice nucleation via collisions with supercooled droplets (58).

Electric charges may also impact the atmospheric lifetime and transport properties of aerosol particles. Motivated by the observed transport of Saharan dust (<2.5 μm) at distances far greater than predicted by models (59, 60), it was recently shown that charges could prolong the lifetime of micron-sized particles (25, 26). The mechanism involves the influence of ambient electric fields on their transport such as those observed in thunderstorms (61) or fair weather (62), allowing dust particles to be carried aloft and resist gravitational settling (63).

In addition to the role that charge may play in the transport and climatic impacts of BWPs, the observation that BWPs are mostly charged, and that most are highly charged, suggests that mitigation efforts such as that proposed by Woo et al. (33) can be highly effective. This can have far-reaching consequences, since the environmental impacts of BWPs extend beyond the atmosphere to aquatic systems (64) and soils (65). Our observations also imply a sizeable dependence of charged particle number on brake pad type, motivating the need to determine the relationship between particle electrical properties and brake pad composition. Understanding the relationships between BWP size, charge state, and key state variables such as braking torque and rotor surface temperature can ultimately lead to the development of true zero-emission vehicles that would lessen impacts of motor vehicles on human health and the environment.

## Materials and Methods

### Section 1: Brake Dynamometer.

The custom-built dynamometer used in this study (schematic provided in *SI Appendix*, Fig. S1) employed a heavy-duty metal-working lathe to rotate a G3000 rotor (BrakeBest, model 96211RGS), which was capable of achieving the torque produced in real-world braking. The disk brake caliper was a common model (Kodiak, model 225) for which a large variety of brake pads are commercially available. Two brake pads were tested: ceramic (Kodiak, model DBC-225) and semi-metallic (BrakeBest, model MKD289). Braking force was applied using an electric over hydraulic brake actuator (Hydrastar, model HBA-12) and a brake controller (Tekonsha, model PowerTrac), the latter of which was modified to accept computer control of braking force and time. An 87 L aluminum chamber enclosed the brake system to allow for clean, particle-free air to be introduced using a purge air generator (Parker Balston, model 75-62) with a downstream HEPA capsule filter (TSI, part # 1602051), thus isolating sampled emissions from background air. Also housed in the chamber were sensors for monitoring chamber relative humidity and temperature (Vaisala, model HMP-44), an infrared non-contact temperature sensor for measuring the temperature of the rotor surface (Omega, model OS301-HT), a pressure sensor for monitoring the pneumatic fluid pressure (AiM, MC-327), and a torque sensor for monitoring the torque applied to the brake caliper (ATO, model ATO-TQS-S01). DOT 3 brake fluid (O’Reilly, part 72120) was used for the braking system.

Braking was simulated by rotating the brake rotor at constant speed and applying a step-function pattern of braking pneumatic pressure to the caliper. This methodology is similar to that adopted by Zessinger et al. (66). This approach allowed us to achieve characteristic operating points such as braking torque and rotor temperature in a consistent manner that can be successfully reproduced over time. The rotor speed was maintained at 173 rpm for the duration of each experiment. For these experiments, a pneumatic pressure of 1,400 kPa, corresponding to a measured braking torque of 120 N·m, was applied to the brake caliper for a duration of 8 s, followed by a cruising phase (no braking) of 45 s. This pattern was repeated for 1 to 2 h, heating the rotor surface to a temperature of ∼130 °C. Each brake pad underwent a bedding procedure using the same braking cycles described above, reaching rotor temperatures up to ∼200 °C.

Particles inside the chamber were counted and sized using an aerodynamic particle sizer (APS; TSI, Inc., model 3321), which measured over an aerodynamic diameter range of 500 to 22,000 nm, and a scanning mobility particle sizer (SMPS) consisting of a differential mobility analyzer (DMA, TSI, Inc., model 3081) coupled to a mixing condensation particle counter (MCPC; Brechtel Manufacturing, Inc., model 1720), which measured an electrical mobility diameter range of 10 to 900 nm. The chamber was continuously flushed with particle-free purge air at a flow rate of 35 L min^−1^, and interior surfaces were periodically cleaned with vacuum suction and wiped with isopropyl alcohol to minimize artifacts caused by particle resuspension.

### Section 2: Charged Fraction Measurement.

The fraction of charged BWPs was determined using two water-based CPCs (Aerosol Devices, Inc., model MAGIC 210) measuring particle number concentrations in parallel (size range5 to 2,500 nm), with one CPC measuring downstream of a home-built electrostatic precipitator and the other serving as a bypass counter for measuring total particle concentration. The electrostatic precipitator served as a deposition sink for charged aerosol, allowing only electrically neutral particles to be transmitted. It consisted of a 3-mm diameter electrically isolated rod located on the axis of a 150-mm length of stainless steel straight tubing with a 7.5-mm inner diameter. A voltage of 1,200 V was applied to the inner rod and the outer tube was grounded. This voltage was chosen by taking into account the precipitator dimensions as well as the inlet flow rate (0.3 L min^−1^) and was experimentally confirmed by varying the voltage to a point where the particle concentration passing through the precipitator was stable. The charged particle fraction was therefore determined by subtracting the number concentrations measured by the two CPCs and dividing by the bypass concentration.

### Section 3: Charge State Measurement.

The electrical charge state of brake wear aerosol was probed in a series of experiments using a tandem differential mobility analyzer (TDMA) system, in a manner similar to what has been reported previously (50). Before each experiment, the brakes were subjected to 1 h of braking to reach the condition at which charged particle concentrations reached steady state. *SI Appendix*, Fig. S8 depicts the electrical mobility distributions and braking conditions during these warm-up periods. The experimental layout is depicted in *SI Appendix*, Fig. S1. Briefly, a DMA (TSI, model 3081) was set to a single voltage to select particles of a particular electrical mobility (e.g., an equivalent mobility diameter of 50 nm if the particles are considered only singly charged). The mobility-selected aerosol is then conditioned to a known charge state by passing the sample stream through a bipolar neutralizer developed by the Particle Technology Lab (PTL) at the University of Minnesota as described by Jiang et al. (67). A second DMA (TSI model 3081) then scanned over the full mobility diameter range (10 to 730 nm), whereafter particles were counted with an MCPC. Data were inverted and charge state distributions calculated using the methods described in Section 4. The bipolar neutralizer was removed before each experiment to observe the transfer function of the first DMA. The results of these measurements are depicted in *SI Appendix*, Fig. S6. Experiments were repeated to obtain charge state distributions for both negatively and positively charged particles with electrical mobility diameters ranging from 35 to 95 nm.

Additionally, to examine the charged particle distributions across a broader range of mobilities, the electrical mobility distributions for both positively and negatively charged BWPs were obtained by using two DMA systems consisting of model 3081 DMAs (TSI, Inc.) and MCPCs operating in parallel with high voltage power supplies of opposing polarity. Both systems were sampled in the absence of bipolar neutralizers to ensure that only ambient charged particle size distributions were obtained.

An aerosol electrometer (Ioner, model EL-5030) was used to monitor the aerosol current generated from braking. This measurement provided the overall magnitude of net charge flow across a cluster and particle size range beyond that of our mobility sizing instrumentation.

### Section 4: Average Charge State Calculation.

Using the TDMA data (Section 3), we determined the total number-size distribution, $n(d_{p})$, by inverting the data with the Washington State University SMPS Data Inversion Toolkit written in Igor Pro (Wavemetrics, Inc.). If sampled particles are multiply charged, this distribution would consist of the superposition of individual transfer functions in diameter space, $\Omega_{d}\left(d_{p,i},i\right)$, where $d_{p,i}$ is the mobility diameter of a particle with i elementary charges:

[1] $$\begin{array}{c} n\left(d_{p}\right)=\sum_{i}h_{i}\cdot \Omega_{d}\left(d_{p,i},i\right), \end{array}$$

$h_{i}$ is the height of the transfer function for particles with i elementary charges. The applied DMA voltages in these experiments were above the range at which diffusional broadening is significant (68). In addition, measurements were over a size range for which the size resolution is mostly determined by the ratio of aerosol-to-sheath flow rates and the transfer function in diameter space $\Omega_{d}$ has a triangular functional form as derived by Knutson and Whitby (69).

Values of $h_{i}$ in Eq. **1** were determined using Levenberg–Marquardt least-squares fitting algorithm in Igor Pro. For the lower charge states, 1≤i≤5, we could directly fit $h_{i}$ to the size distribution. For higher charge states, i> 5, transfer functions overlapped and could not be fitted individually. Therefore, we represented the height of the transfer function as $h_{i}=h_{l}\cdot LogNormal\left(d_{p};d_{p,geo},\sigma_{\mathrm{geo}}\right)$, where $h_{l}$ the height scaling of the log-normal distribution with the location $d_{p,geo}$ and distribution parameter $\sigma_{\mathrm{geo}}$. The individual heights $h_{i}$ represent the occurrence of the ith-charge for the selected mobility $Z^{*}$. With this, we calculated the average number of charges for the corresponding equivalent diameter. The uncertainty of the calculated charge state was analyzed with a Monte-Carlo approach, in which the individual $h_{i}$ were randomly adjusted within the uncertainty range provided by the fit and the charge state recalculated.

## Supplementary Material

Appendix 01 (PDF)

## Data Availability

Comma delimited text files data have been deposited in Data for: Automotive braking is a source of highly charged aerosol particles (https://doi.org/10.5061/dryad.cfxpnvxc3) (70).
