# Supplementary material for: Automotive braking is a source of highly charged aerosol particles
Source: Proc Natl Acad Sci U S A. 2024 Mar 11;121(13):e2313897121. doi: 10.1073/pnas.2313897121 (PMC10990126; doi:10.1073/pnas.2313897121)
Supplement: Supplementary file 1 — Appendix 01 (PDF) [file pnas.2313897121.sapp.pdf]

Supporting Information for

Automotive braking is a source of highly charged aerosol particles

Adam E. Thomas, Paulus S. Bauer, Michelia Dam, Veronique Perraud, Lisa M. Wingen, James N. Smith

James N. Smith
E-mail: jimsmith@uci.edu

This PDF file includes:

Figs. S1 to S10

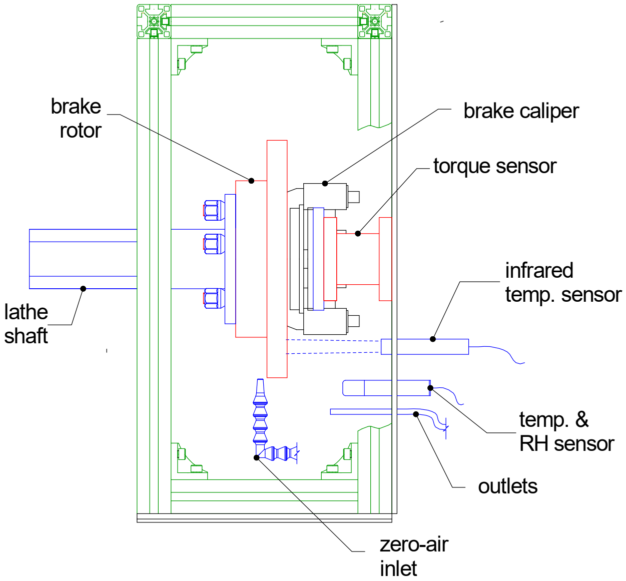

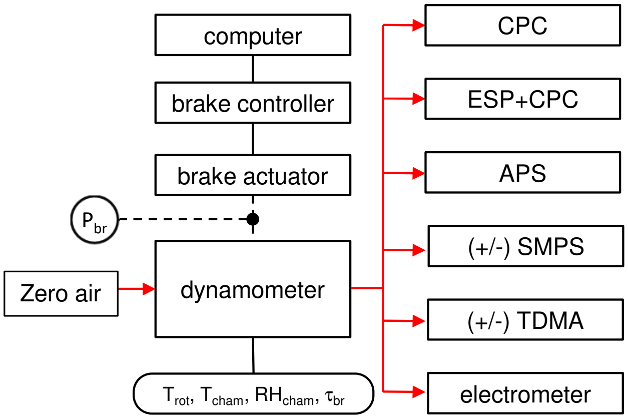

Fig. S1. (Top) Schematic of brake dynamometer. (Bottom) Experimental overview schematic for measurements reported. Acronyms used: Brake pressure (P_{br}), Condensation Particle Counter (CPC), ElectroStatic Precipitator (ESP), Scanning Mobility Particle Sizer (SMPS), Tandem Differential Mobility Analysis (TDMA).

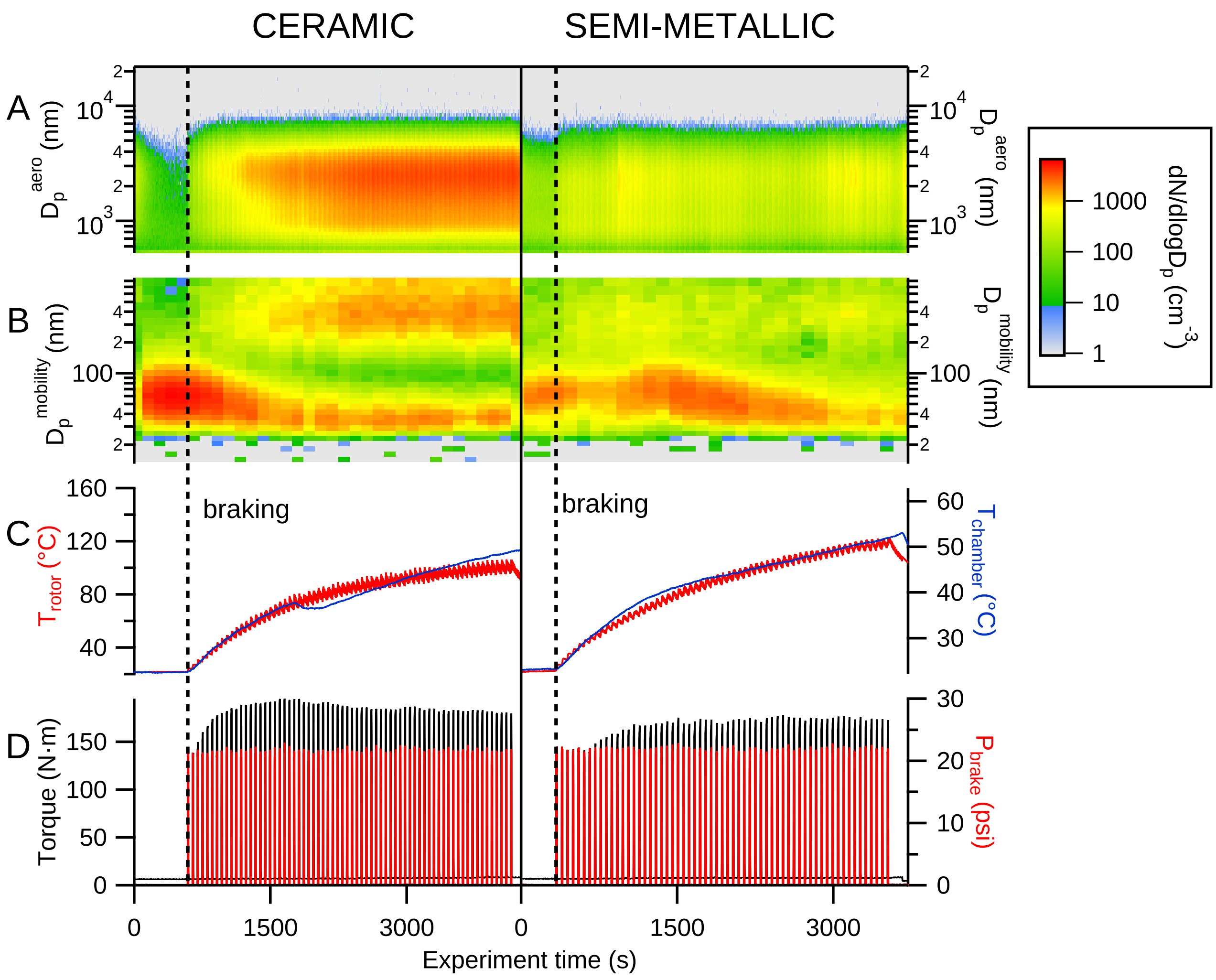

Fig. S2. BWP size distributions with dynamometer (i.e., chamber) parameters during experiments with ceramic (left) and semi-metallic (right) brake pads. (A) APS size distribution, acquiring particles with aerodynamic diameters (D_p^{aero}) 500 and 22000 nm. (B) SMPS size distribution, scanning particles with electrical mobility diameters ($D_p^{mobility}$) 10 - 900 nm. Braking conditions depicted for (C) brake rotor (T_rotor, in red) and chamber ($T_chamber$, in blue) temperature, and (D) brake torque (in black) and pressure (P_brake, in red).

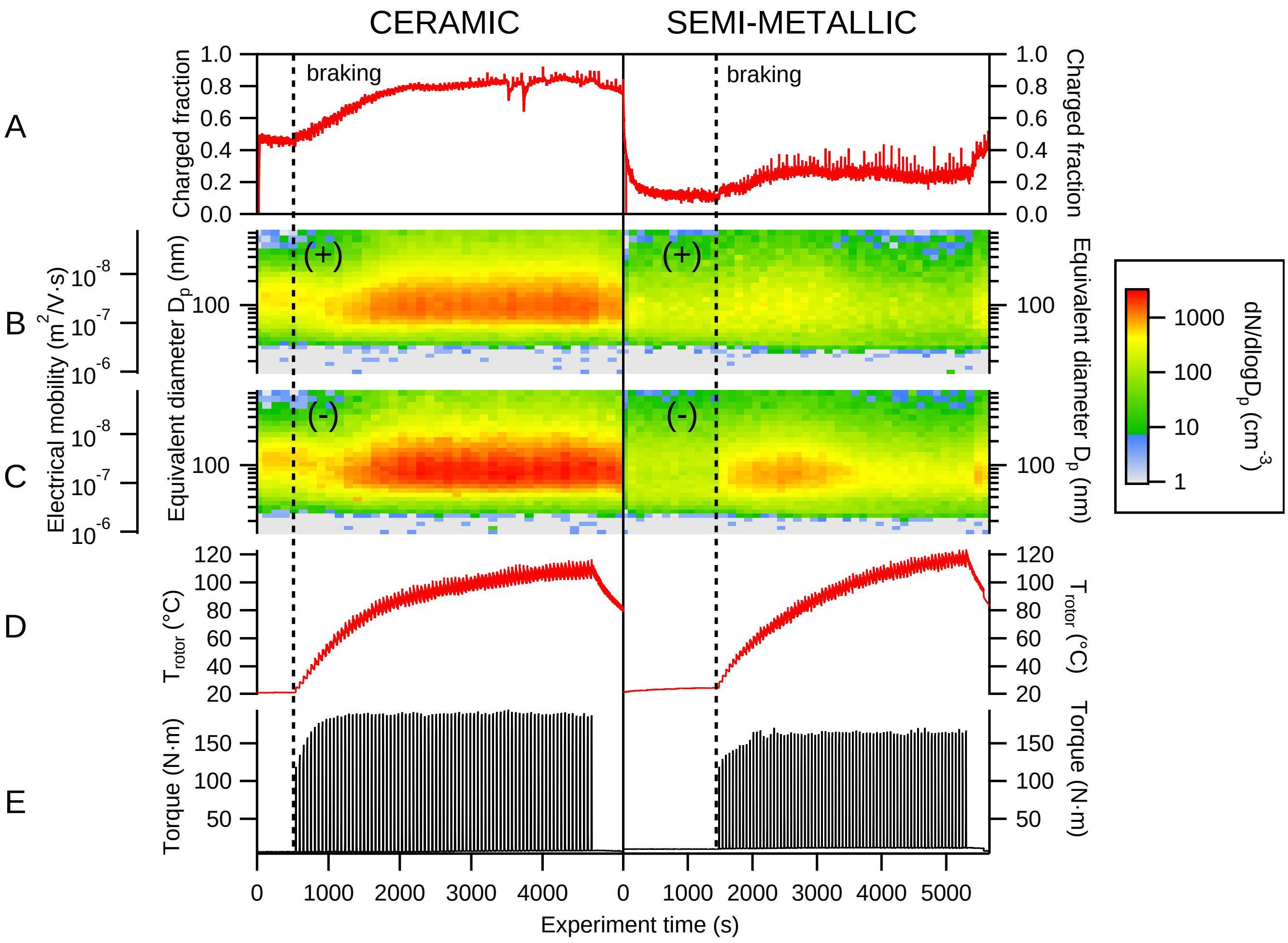

Fig. S3. Charged particle fraction and dual SMPS measurements shown with chamber parameters over course of entire experiment time. (A) Charged particle fractions for experiments with ceramic (left) and semi-metallic (right) brake pads. Fractions shown in red are smoothed data while the variation in the raw data is shown in a lighter shade. The dashed lines mark the initiation of braking. (B-C) SMPS electrical mobility distributions of naturally charged BWPs with (A) positive and (B) negative charges. (D) Rotor temperature and (E) torque over experiment time.

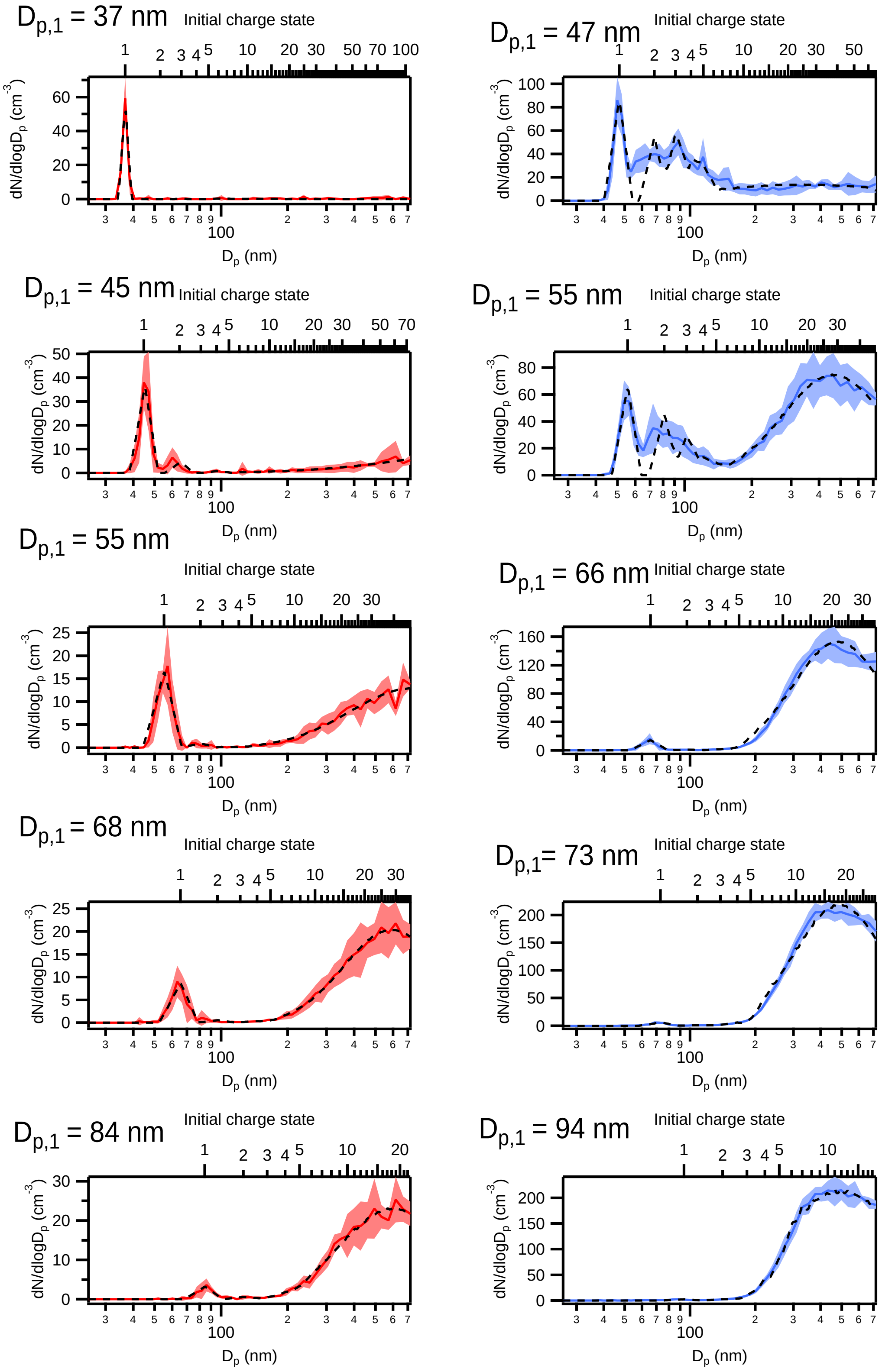

Fig. S4. Full TDMA data set for ceramic brake pad BWPs of positive (left column, in red) and negative (right column, in blue) charge states. Charge state distribution scans are labeled by the singly charged electrical mobility diameter $(D_p, 1)$ as defined for the average charge state calculation. The dotted lines indicate the model results. Shaded areas correspond to $\pm$ the standard deviation of multiple measurements.

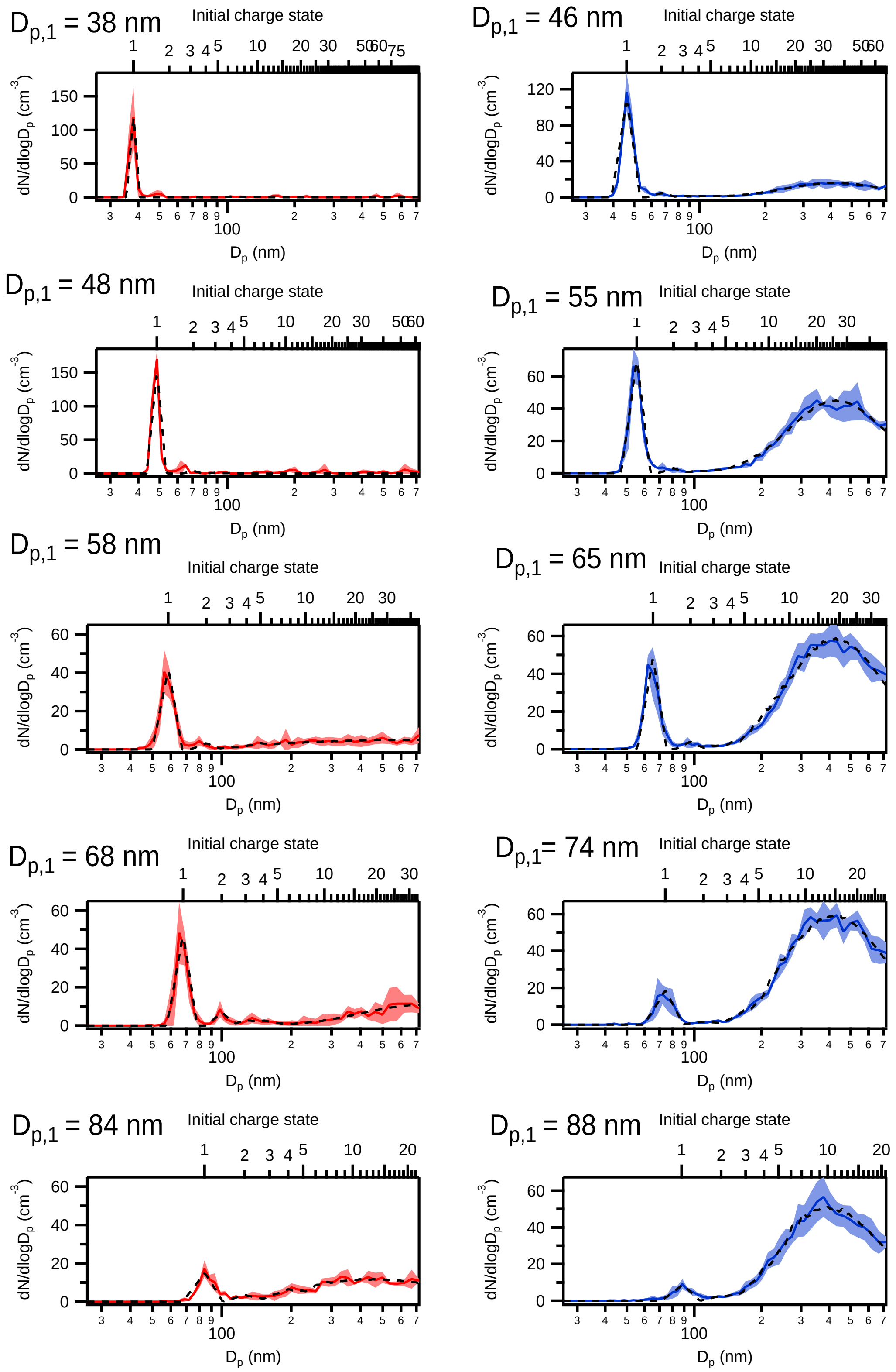

Fig. S5. Full TDMA data set for semi-metallic brake pad BWPs of positive (left column, in red) and negative (right column, in blue) charge states. Charge state distribution scans are labeled by the singly charged electrical mobility diameter $(D_p, 1)$ as defined for the average charge state calculation. The dotted lines indicate the model results. Shaded areas correspond to $\pm$ the standard deviation of multiple measurements.

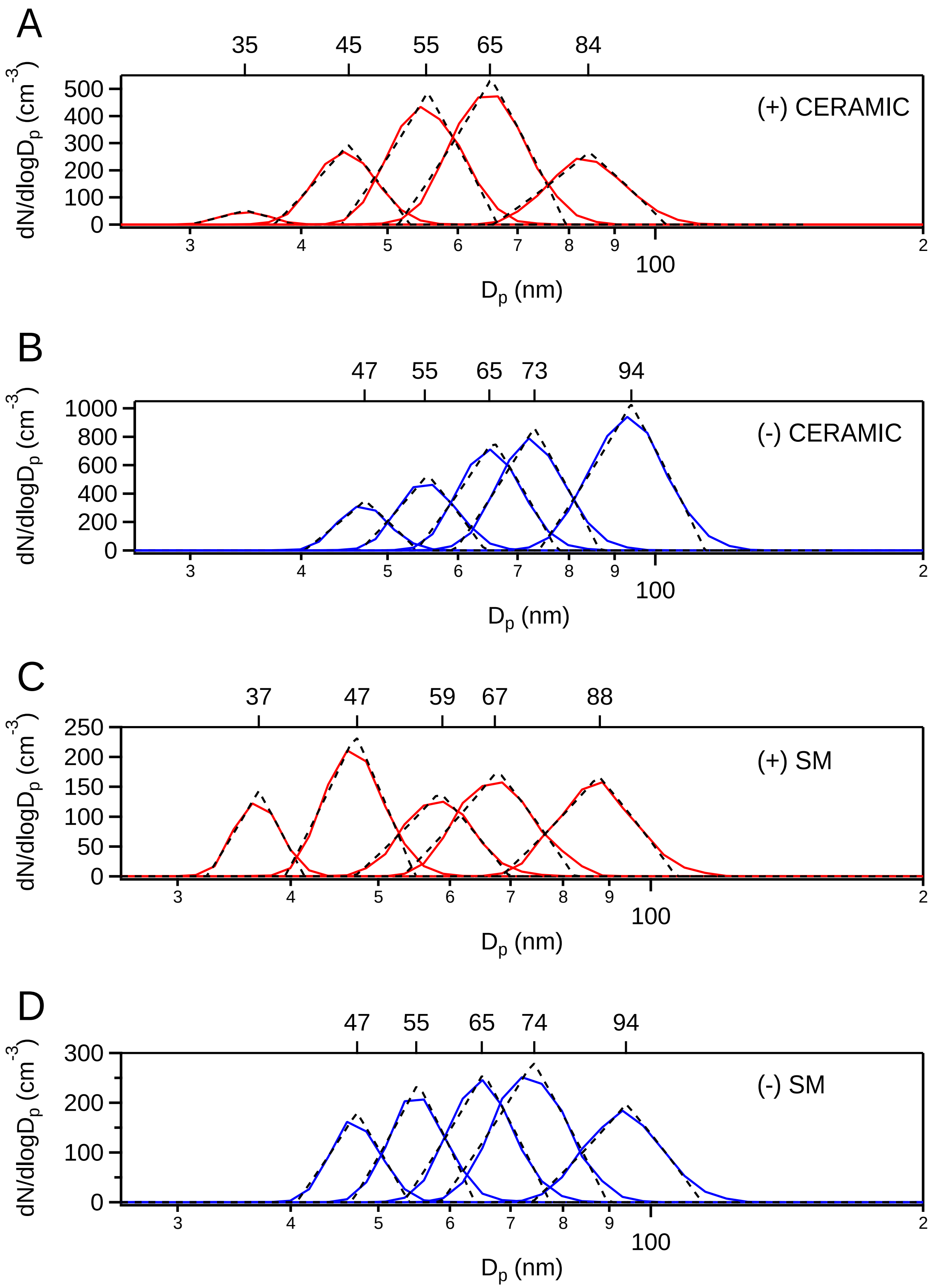

Fig. S6. TDMA scans showing the transmission of positively (in red) and negatively (in blue) charged particles through DMA1 (TSI 3081) at each selected equivalent mobility diameter for (A-B) ceramic BWPs and (C-D) semi-metallic BWPs. Dashed black lines indicate the model fit results. Note that some of the peak diameters differ from those used in the charge state fits due to the diameter bin width used for these measurements (2 nm) as well as the effect from averaging multiple scans.

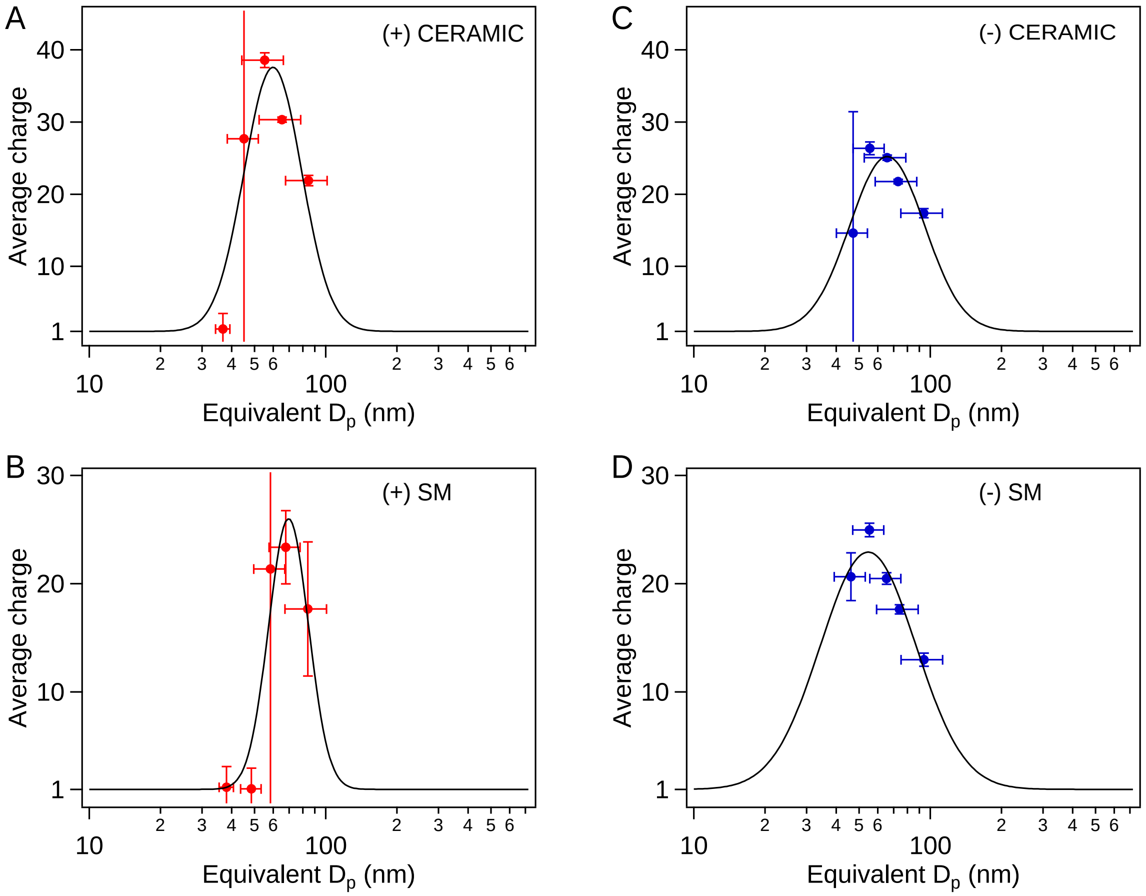

Fig. S7. Average charge state as a function of equivalent singly charged mobility diameter (referred to here as simply "Equivalent Dp") selected for TDMA analysis, depicted for positively charged particles (left column, in red) measured from (A) ceramic and (B) semi-metallic brake pads, and negatively charged particles (right column, in blue) measured from (C) ceramic and (D) semi-metallic brake pads. Y-axis errors are ± the percent uncertainty in the average charge calculation, while the X-axis errors are ± the DMA resolution used to fit each charge state distribution. The black trace in each plot is the log-normal fitting of the data extrapolated over the SMPS scanning range.

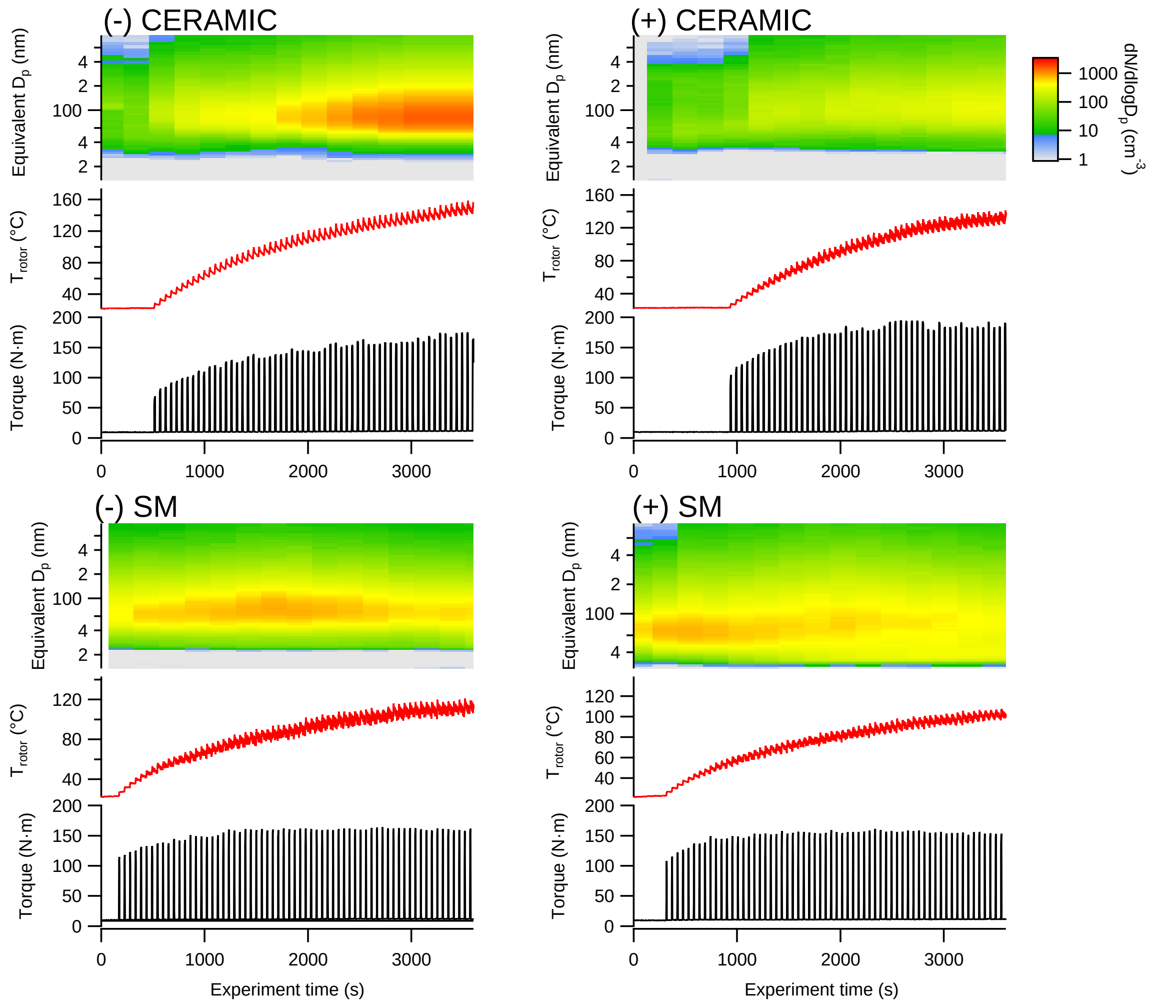

Fig. S8. Braking conditions and electrical mobility distributions (no neutralizer attached) during the 1-hr warm-up periods before the TDMA measurement of charged particles (+/-) from ceramic and semi-metallic (SM) brakes.

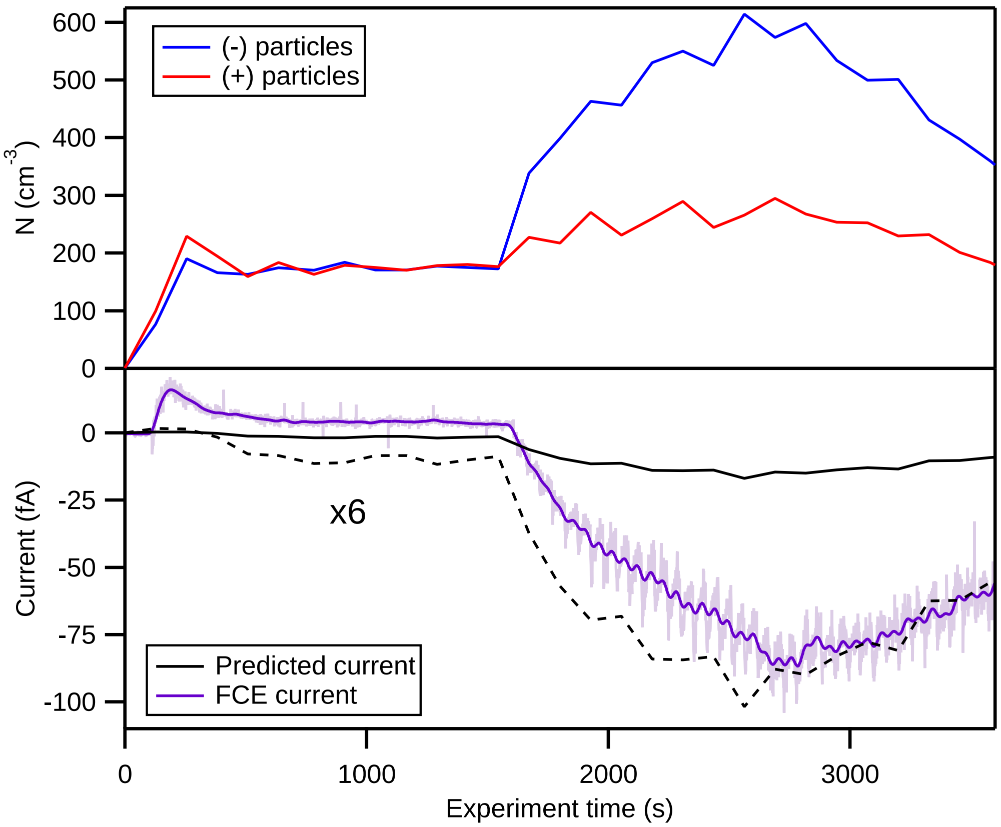

Fig. S9. (A) Concentrations of negatively (blue) and positively (red) charged particles and (B) aerosol currents for semi-metallic brake pads. Predicted current (solid black) obtained from SMPS and TDMA measurements is plotted with an amplified version (dashed black) to compare the waveform against the FCE current (purple). The light purple trace indicates the raw signal from the FCE while dark purple is smoothed data.

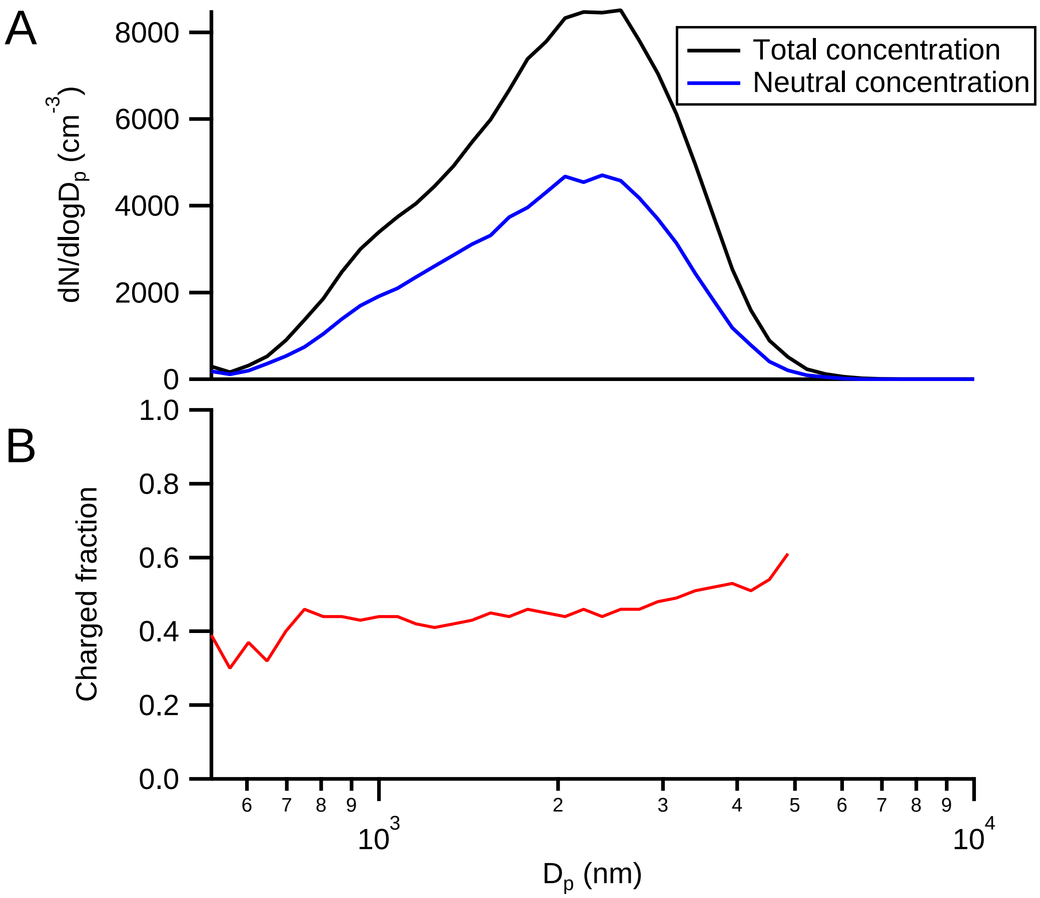

Fig. S10. (A) Size distribution from the APS during an experiment with ceramic brakes taken downstream of an electrostatic precipitator. Bypass particle concentration (total concentration) is measured when the precipitator is off (0 V) while the neutral particle concentration is measured while on (1200 V). (B) Qualitative charged fraction as a function of aerodynamic particle diameter. Results are not quantitative since the size of the precipitator used was not optimized for the APS flow rate (5 LPM).
